# Serial cross-sectional assessment of glycaemic and blood pressure profiles at a KwaZulu-Natal tertiary hospital: A 5-year analysis (2021–2025)

**DOI:** 10.4102/safp.v68i1.6332

**Published:** 2026-09-04

**Authors:** Somasundram Pillay

**Affiliations:** 1 Department of Internal Medicine, School of Medicine, College of Health Sciences, University of KwaZulu-Natal, Durban, South Africa; 2 Department of Internal Medicine, Victoria Mxenge Hospital, Durban, South Africa

**Keywords:** Diabetes mellitus, Hypertension, HbA1c, Dysglycaemia, Multimorbidity, HIV, South Africa

## Abstract

**Background:**

Diabetes, hypertension and HIV-associated multimorbidity contribute substantially to South Africa’s cardiometabolic disease burden. We described five years of screening for dysglycaemia, glycaemic control, blood pressure control and retroviral disease (RVD)-associated multimorbidity at a KwaZulu-Natal tertiary hospital.

**Methods:**

Annual single-day cross-sectional audits were conducted from 2021 to 2025 among outpatient attendees and volunteering hospital staff. Participants underwent point-of-care testing (POCT) of glycated haemoglobin (HbA1c) and seated brachial blood pressure measurement. Dysglycaemia was defined as HbA1c ≥ 5.7% in participants without known diabetes, glycaemic control in known diabetes as HbA1c < 7.0%, and blood pressure control as < 140/90 mmHg. Proportions were reported with Wilson 95% confidence intervals.

**Results:**

Among 411 records, known diabetes was present in 98/407 participants (24.1%; 95% CI 20.2–28.5) and known hypertension in 149/334 (44.6%; 95% CI 39.4–50.0). Among participants without known diabetes, 138/298 had dysglycaemia (46.3%; 95% CI 40.7–52.0), including 49/298 with diabetes-range HbA1c (16.4%; 95% CI 12.7–21.1). Glycaemic control was achieved in 37/98 participants with known diabetes (37.8%; 95% CI 28.8–47.6). In years with reconcilable data, 41/107 participants with known hypertension achieved blood pressure control (38.3%; 95% CI 29.7–47.8). In 2025, RVD-positive status was recorded in 29/101 participants (28.7%; 95% CI 20.8–38.2), with overlap between RVD, diabetes and hypertension.

**Conclusion:**

This selected hospital-based population had a high burden of dysglycaemia and suboptimal diabetes and blood pressure control. RVD-associated multimorbidity supports standardised screening, confirmatory testing and integrated chronic disease follow-up.

**Contribution:**

This 5-year serial screening analysis demonstrates a substantial burden of dysglycaemia, incomplete glycaemic control and suboptimal BP control in a selected hospital-based screening population. RVD-associated cardiometabolic multimorbidity was observed in 2025, supporting the need for standardised opportunistic screening, confirmatory testing pathways and integrated chronic disease follow-up within public-sector services.

## Introduction

Diabetes mellitus and hypertension are major contributors to morbidity, premature mortality and health-system burden globally, with the fastest growth occurring in low- and middle-income countries. The International Diabetes Federation estimates that 537 million adults were living with diabetes in 2021, with Africa projected to experience the greatest proportional increase by 2045.^1^ Hypertension affects more than 1 billion people worldwide and remains a leading modifiable risk factor for cardiovascular disease, stroke, heart failure and chronic kidney disease.^2^ In sub-Saharan Africa, urbanisation, dietary transition, reduced physical activity and population ageing have accelerated the dual burden of diabetes and hypertension.^3,4^

A major challenge in the African region is delayed detection. A substantial proportion of people with diabetes remain undiagnosed, and many individuals with established diabetes or hypertension do not achieve recommended treatment targets.^1,4^ These gaps are clinically important because delayed diagnosis and suboptimal control increase the risk of microvascular disease, cardiovascular events, kidney disease and premature mortality. In South Africa, where public-sector services manage a high burden of chronic disease, opportunistic screening within healthcare settings may help identify individuals requiring confirmatory testing, counselling, treatment intensification and linkage to longitudinal care.

South African primary-care guidance defines adult diabetes using recognised glucose-based diagnostic thresholds, including symptoms with random plasma glucose ≥ 11.1 mmol/L, fasting plasma glucose ≥ 7.0 mmol/L or 2-h oral glucose tolerance test glucose ≥ 11.1 mmol/L.^5^ In asymptomatic individuals identified through screening, diagnosis requires confirmation with two positive tests on separate days.^5^ Current South African guidance also emphasises routine blood pressure (BP) measurement in diabetes care and uses a treatment target around 140/90 mmHg for general cardiometabolic risk reduction.^5,6^ Therefore, abnormal findings from a single awareness-day screening encounter should be interpreted as screen-positive results requiring confirmation and linkage to care, rather than as definitive new diagnoses.

The intersection between communicable and non-communicable diseases further complicates cardiometabolic risk in South Africa. People living with human immunodeficiency virus (HIV) are increasingly affected by metabolic and cardiovascular comorbidities as survival improves with antiretroviral therapy.^7,8^ Chronic immune activation, inflammation, antiretroviral-related metabolic effects, weight gain, dyslipidaemia, insulin resistance and vascular ageing may all contribute to this risk profile.^7,8,9^ Meta-analytic evidence has examined associations between antiretroviral therapy and diabetes risk, although causality and magnitude of effect vary across study designs and populations.^10^ Similarly, increased BP and hypertension risk among people receiving antiretroviral therapy have been described in systematic review evidence.^11^ These data support an integrated approach to HIV and non-communicable disease screening, particularly in high HIV-burden settings such as KwaZulu-Natal.

Single-day hospital-based screening audits provide pragmatic real-world snapshots of glycaemic status, BP control and multimorbidity among people already interacting with the health system. Point-of-care HbA1c testing and BP measurement can rapidly identify abnormal results and control gaps; however, screening alone is insufficient unless paired with confirmatory testing, treatment review and structured follow-up. Serial cross-sectional data from tertiary hospitals in South Africa remain limited, particularly where glycaemic, BP and HIV or RVD-associated multimorbidity data are assessed together.

Since 2021, annual cardiometabolic screening audits have been conducted at Victoria Mxenge Hospital, a public-sector tertiary hospital in KwaZulu-Natal. These audits provide repeated cross-sectional snapshots of known diabetes, known hypertension, HbA1c-defined dysglycaemia and cardiometabolic control in a selected hospital-based screening population. From 2025, HIV or RVD status was incorporated into the audit, allowing description of HIV-associated cardiometabolic multimorbidity within the same screening framework.

The present study reports a 5-year serial cross-sectional analysis of annual screening audits conducted between 2021 and 2025. The objectives were to quantify the prevalence of known diabetes and known hypertension, determine the proportion of participants without known diabetes who had HbA1c-defined dysglycaemia, assess glycaemic and BP control among participants with established disease, and describe HIV or RVD-associated multimorbidity in the 2025 cohort. By providing source-reconciled serial data from a KwaZulu-Natal tertiary hospital, this study aims to inform opportunistic screening, confirmatory testing pathways and integrated chronic disease follow-up within South Africa’s public-sector health system.

## Research methods and design

### Study design and setting

This study comprised five consecutive serial cross-sectional screening audits conducted annually between 2021 and 2025 at Victoria Mxenge Hospital, a public-sector tertiary hospital in KwaZulu-Natal, South Africa. Each audit was performed on a single designated screening day as part of institutional cardiometabolic awareness activities. The present analysis aggregates these independent annual cross-sectional datasets into a 5-year descriptive series.

### Participants

People attending outpatient services on the screening day and hospital staff who volunteered for screening were included. The sample was a non-probability convenience sample of people already engaged with, or employed by, the health system. Age eligibility was not consistently documented across source files. Participation was voluntary and based on verbal informed consent in keeping with institutional quality-improvement practice.

Following source reconciliation, 411 completed screening records were available for analysis: 74 in 2021; 71 in 2022; 65 in 2023; 94 in 2024; and 107 in 2025.

### Source data reconciliation and quality control

Source data were reconciled before analysis. Participant-level data were prioritised over secondary narrative summaries where discrepancies were identified. The 2025 handwritten datasheets contained numbered rows up to 110; however, rows 108–110 were blank, giving 107 completed 2025 records. The 2024 blood-pressure-control estimate was removed from pooled BP-control analysis and treated as not estimable, because the source report used conflicting BP thresholds and subgroup percentages. No imputation was performed. All analyses used outcome-specific denominators, and missing observations were excluded only from the relevant analysis.

### Data collection

A standardised data-capture sheet was used annually. The following variables were recorded where available: age, sex, prior diagnosis of diabetes mellitus, prior diagnosis of hypertension, HIV or RVD status in 2025, random capillary glucose, point-of-care HbA1c, brachial BP and pulse rate.

### HbA1c measurement

HbA1c was measured using a handheld point-of-care analyser operated by trained nursing staff. Point-of-care HbA1c testing was performed using the BioHermes® handheld POCT device (Wuxi BioHermes Biomedical Technology Co., Jiangsu, China), which uses boronate affinity chromatography for HbA1c quantification. HbA1c values were used to classify glycaemic status and glycaemic control according to the operational definitions below.

### Blood pressure measurement

Blood pressure and pulse rate were measured using the Mindray^®^ VS-800 vital signs monitor (Mindray Medical International, Shenzhen, China), which uses automated oscillometric technology for non-invasive BP measurement. Participants were seated and rested for at least 5 min before measurement. If the initial reading was elevated, defined as systolic BP ≥ 140 mmHg and/or diastolic BP ≥ 90 mmHg, a repeat measurement was obtained, and the lower value was recorded. Appropriate cuff sizing was selected based on arm circumference.

Because repeat BP measurement was performed only when the first reading was elevated, many participants had a single retained reading. This differs from preferred repeated-measurement practice and may introduce measurement error or regression-to-the-mean effects.

### Random capillary glucose

Random capillary glucose was collected as an ancillary same-day clinical measure and was summarised descriptively where participant-level data were available. It was not used to assign glycaemic categories.

### Definitions

Known diabetes was defined as a prior self-reported or documented diagnosis of diabetes mellitus. Known hypertension was defined as a prior self-reported or documented diagnosis of hypertension.

Among participants without known diabetes, HbA1c-defined dysglycaemia was defined as HbA1c ≥ 5.7%, comprising both the pre-diabetes range and diabetes-range HbA1c values. Screen-positive diabetes-range HbA1c was defined as HbA1c ≥ 6.5% among participants without known diabetes. These participants were not classified as having confirmed diabetes unless confirmatory diagnostic testing was performed after the screening encounter.

Among participants with known diabetes, glycaemic control was defined as HbA1c < 7.0%. Blood pressure control among participants with known hypertension was defined as BP < 140/90 mmHg. RVD-positive status referred to recorded RVD status from self-report or existing clinical record. No audit-day HIV testing was documented.

Multimorbidity was defined descriptively as the coexistence of two or more recorded chronic conditions among diabetes mellitus, hypertension and RVD-positive status.

### Outcome measures

The primary outcomes were:

- Prevalence of known diabetes.
- Prevalence of known hypertension.
- Prevalence of HbA1c-defined dysglycaemia among participants without known diabetes.
- Proportion of participants with known diabetes achieving HbA1c < 7.0%.
- Proportion of participants with known hypertension achieving BP < 140/90 mmHg.
- RVD-associated cardiometabolic multimorbidity in the 2025 cohort.

### Statistical analysis

Categorical variables are reported as *n/N* (%) with Wilson 95% confidence intervals (CIs). Continuous variables are summarised as median, interquartile range and range. Exploratory Cochran–Armitage trend tests were used only where definitions were sufficiently consistent. Fisher exact tests compared 2025 RVD-positive and RVD-negative groups. All analyses used complete-case denominators; no imputation was performed.

### Ethical considerations

This study was conducted as a service evaluation and quality-improvement audit using anonymised screening data. No identifiable patient-level information is reported. Ethics approval was obtained from the University of KwaZulu-Natal Biomedical Research Ethics Committee (BREC/00009771/2025).

## Results

### Participant characteristics

Across five annual screening audits, 411 completed screening records were available for analysis: 74 in 2021; 71 in 2022; 65 in 2023; 94 in 2024; and 107 in 2025. Demographic data were not available for the 2021 audit. Participant-level age and sex data were available for 2022; 2023; and 2025, while the 2024 report provided aggregate demographic data. The annual cohorts were predominantly female in all years with available sex data. Median age ranged from 46 years in 2022 to 55 years in 2023, although recorded age ranges included some younger participants, reflecting inconsistent age eligibility documentation across source files (Table 1).

**TABLE 1:** Participant characteristics by audit year.

Year	Completed records	Age (years)	Median (IQR)	Range	Female		
					*n*	*N*	%
2021	74	Not available	-	-	Not available	Not available	-
2022	71	46	33–54	14–81	48	71	67.6
2023	65	55	44–64	1–85	47	65	72.3
2024	94	50	-	12–86	65	94	69.1
2025	107	51	41–60	13–89	74	107	69.2

IQR, interquartile range.

### Annual clinical and metabolic indicators

Known diabetes was recorded in 98/407 participants across the 5-year period (24.1%; 95% CI 20.2–28.5). Known hypertension was recorded in 149/334 participants from years with available hypertension-status data (44.6%; 95% CI 39.4–50.0). Among participants without known diabetes, HbA1c-defined dysglycaemia was present in 138/298 (46.3%; 95% CI 40.7–52.0), including screen-positive diabetes-range HbA1c in 49/298 (16.4%; 95% CI 12.7–21.1).

Among participants with known diabetes, glycaemic control was achieved in 37/98 (37.8%; 95% CI 28.8–47.6). In years with reconcilable blood-pressure-control data, 41/107 participants with known hypertension achieved BP < 140/90 mmHg (38.3%; 95% CI 29.7–47.8). The 2024 BP-control estimate was not included in the pooled BP-control analysis because the source report used conflicting BP thresholds and subgroup percentages (Table 2 and Figure 1).

**FIGURE 1:**
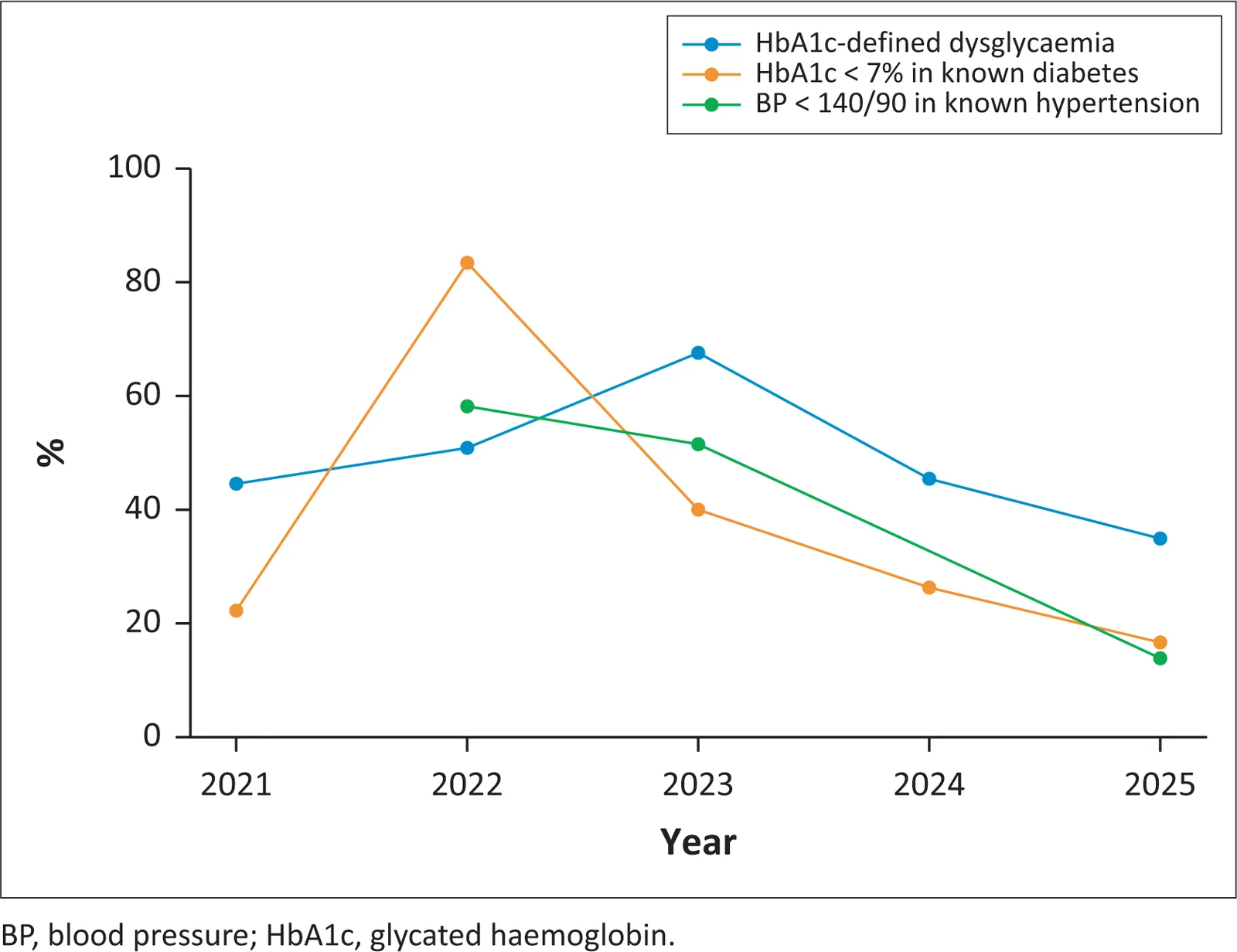
Annual trends in HbA1c-defined dysglycaemia among participants without known diabetes, glycaemic control among participants with known diabetes, and blood pressure control among participants with known hypertension, 2021–2025. Blood pressure control is not shown for 2021 because hypertension or blood pressure-control data were not systematically recorded, and it is not shown for 2024 because the blood pressure-control estimate was not estimable after source reconciliation.

**TABLE 2:** Annual clinical and metabolic indicators from serial screening audits, 2021–2025.

Year	Known diabetes				Known hypertension				HbA1c <7% in known diabetes				HbA1c-defined dysglycaemia†				Screen-positive diabetes-range HbA1c‡				BP <140/90 in known hypertension			
	*n*	*N*	%	CI	*n*	*N*	%	CI	*n*	*N*	%	CI	*n*	*N*	%	CI	*n*	*N*	%	CI	*n*	*N*	%	CI
2021	18	74	24.3, 95	16.0–35.2	-	-	-	-	4	18	22.2, 95	9.0–45.2	25	56	44.6, 95	32.4–57.6	14	56	25.0, 95	15.5–37.7	Not available	Not available	Not available	Not available
2022	18	71	25.4, 95	16.7–36.6	31	71	43.7, 95	32.7–55.2	15	18	83.3, 95	60.8–94.2	27	53	50.9, 95	37.9–63.9	13	53	24.5, 95	14.9–37.6	18	31	58.1, 95	40.8–73.6
2023	25	65	38.5, 95	27.6–50.6	33	65	50.8, 95	38.9–62.5	10	25	40.0, 95	23.4–59.3	27	40	67.5, 95	52.0–79.9	8	40	20.0, 95	10.5–34.8	17	33	51.5, 95	35.2–67.5
2024	19	94	20.2, 95	13.3–29.4	42	94	44.7, 95	35.0–54.7	5	19	26.3, 95	11.8–48.8	30	66	45.5, 95	34.0–57.4	6	66	9.1, 95	4.2–18.4	Not estimable	Not estimable	Not estimable	Not estimable
2025	18	103	17.5, 95	11.3–25.9	43	104	41.3, 95	32.4–51.0	3	18	16.7, 95	5.8–39.2	29	83	34.9, 95	25.6–45.7	8	83	9.6, 95	5.0–17.9	6	43	14.0, 95	6.6–27.3

BP, blood pressure; CI, confidence interval; HbA1c, glycated haemoglobin.

† , HbA1c-defined dysglycaemia was defined as HbA1c ≥ 5.7% among participants without known diabetes;

‡ , Screen-positive diabetes-range HbA1c was defined as HbA1c ≥ 6.5% among participants without known diabetes. This was not interpreted as confirmed diabetes because confirmatory diagnostic testing was not performed during the screening encounter.

Exploratory trend testing showed no clear ordered annual trend in known diabetes prevalence (*p* = 0.139), known hypertension prevalence (*p* = 0.553) or HbA1c-defined dysglycaemia among participants without known diabetes (*p* = 0.118). Screen-positive diabetes-range HbA1c declined across the audit period (*p* = 0.002). Glycaemic target attainment declined from 2022 to 2025 (*p* < 0.001), although interpretation is limited by the small annual denominators and the outlying 2022 estimate. Blood pressure control declined across the years with reconcilable BP-control data (2022, 2023 and 2025; *p* < 0.001).

### Continuous HbA1c, blood pressure and random-glucose distributions

Participant-level continuous data were available for 2022, 2023 and 2025. These data showed skewed distributions and wide ranges for HbA1c, systolic BP, diastolic BP and random capillary glucose. Median HbA1c was highest in 2023 and lowest in 2025. Median systolic BP increased from 126 mmHg in 2022 to 144 mmHg in 2025 (Table 3).

**TABLE 3:** Continuous HbA1c, blood-pressure and random-glucose distributions in years with participant-level data.

Year	HbA1c				SBP				DBP				Random glucose			
	*n*	Median	IQR	Range	*n*	Median	IQR	Range	*n*	Median	IQR	Range	*n*	Median	IQR	Range
2022	71	5.8	5.3–6.3	4.5–13.2	71	126	112–143	87–193	71	74	65–83	52–132	71	5.7	5.0–7.2	4.1–30.5
2023	65	6.2	5.7–7.7	3.9–16.0	64	132.5	120.5–146	92–223	64	77	67.8–84	51–130	65	5.4	4.7–7.6	2.0–23.5
2025	99	5.6	5.1–6.5	3.7–15.9	107	144	128.5–155.5	91–211	107	80	74.5–89	57–145	106	5.8	5.2–6.7	4.1–21.6

SBP, systolic blood pressure; DBP, diastolic blood pressure; IQR, interquartile range; HbA1c, glycated haemoglobin.

### RVD status and cardiometabolic multimorbidity in 2025

RVD status was available for 101/107 completed 2025 records. RVD-positive status was recorded in 29/101 participants (28.7%; 95% CI 20.8–38.2). In complete-case subgroup analysis, known diabetes was similarly distributed between RVD-positive and RVD-negative participants. Known hypertension was numerically more frequent among RVD-positive participants, but the CIs were wide, and the difference was not statistically significant. HbA1c-defined dysglycaemia among those without known diabetes was not higher among RVD-positive participants (Table 4 and Figure 2).

**FIGURE 2:**
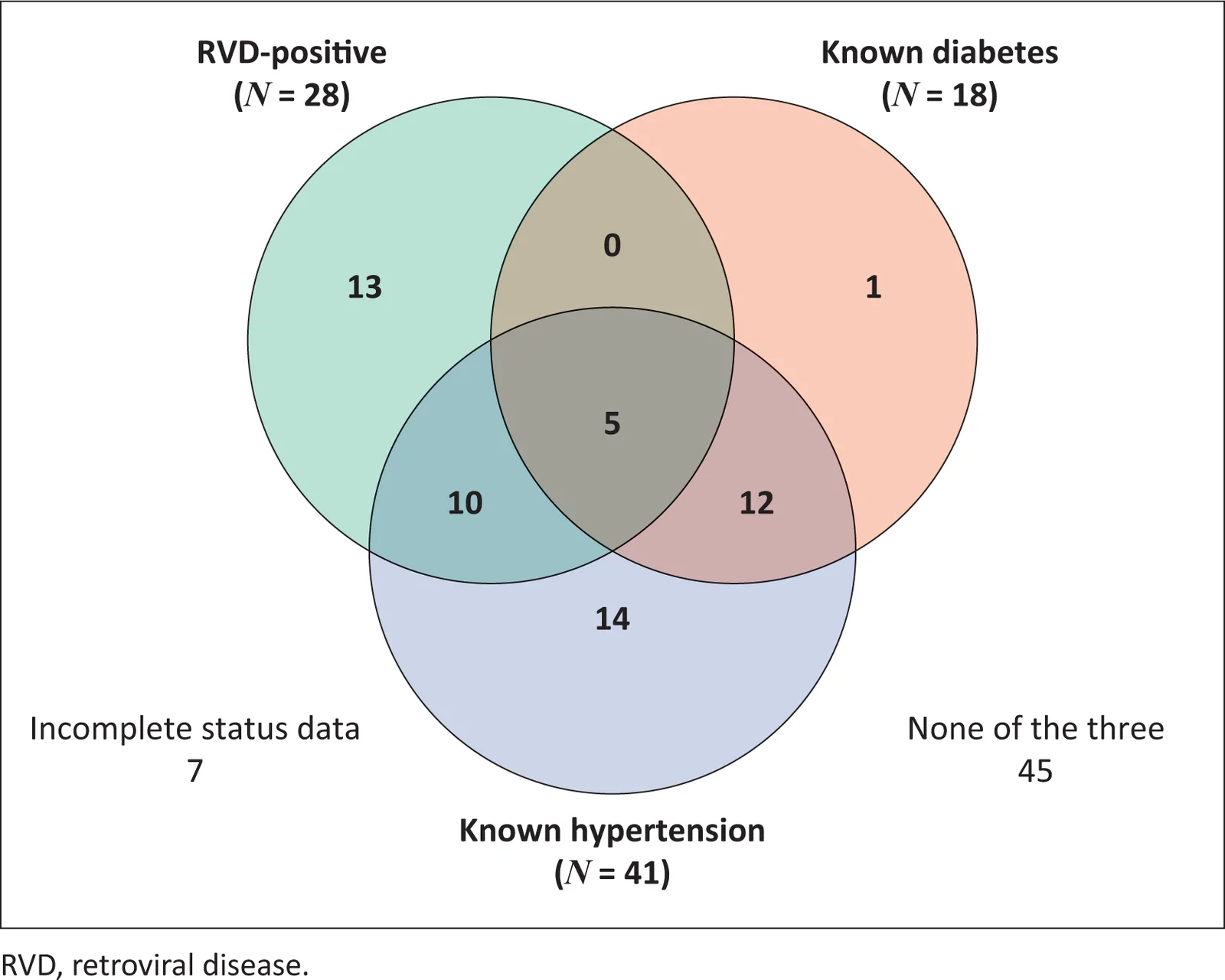
Complete-case overlap of RVD-positive status, known diabetes and known hypertension in the 2025 cohort. The figure is based only on participants with complete data for RVD status, known diabetes and known hypertension. Values represent exclusive region counts. The central overlap represents participants with all three recorded conditions. The seven participants with incomplete status data are not included in the Venn regions.

**TABLE 4:** RVD-stratified cardiometabolic outcomes in the 2025 cohort.

Outcome	RVD-positive				RVD-negative				Odds ratio	Fisher exact *p*
	*n*	*N*	%	95% CI	*n*	*N*	%	95% CI		
Known diabetes	5	28	17.9	7.9–35.6	13	72	18.1	10.9–28.5	0.99	1.000
Known hypertension	16	29	55.2	37.5–71.6	26	72	36.1	26.0–47.6	2.18	0.118
HbA1c-defined dysglycaemia among those without known diabetes	6	23	26.1	12.5–46.5	22	57	38.6	27.1–51.6	0.56	0.316
Screen-positive diabetes-range HbA1c among those without known diabetes	2	23	8.7	2.4–26.8	6	57	10.5	4.9–21.1	0.81	1.000
BP control among those with known hypertension	3	16	18.8	6.6–43.0	2	26	7.7	2.1–24.1	2.77	0.352
Both known diabetes and known hypertension	5	28	17.9	7.9–35.6	12	72	16.7	9.8–26.9	1.09	1.000

BP, blood pressure; CI, confidence interval; HbA1c, glycated haemoglobin; RVD, retroviral disease.

## Discussion

This 5-year series of serial cross-sectional screening audits demonstrates a substantial burden of abnormal glycaemic screening results, incomplete glycaemic target attainment and suboptimal BP control within a selected hospital-based screening population in KwaZulu-Natal. The findings support four key observations: firstly, HbA1c-defined dysglycaemia was common among participants without known diabetes; secondly, glycaemic control among participants with known diabetes was inconsistent across years; thirdly, BP control among participants with known hypertension was poor in years with reconcilable data; and fourthly, RVD-associated cardiometabolic multimorbidity was present in the 2025 cohort, although subgroup comparisons were imprecise.

### Dysglycaemia and screen-positive diabetes-range HbA1c

In the source-reconciled pooled analysis, 138/298 (46.3%; 95% CI 40.7–52.0) participants without known diabetes had HbA1c-defined dysglycaemia, with marked annual variation. This replaces the previous statement that ‘more than half’ of individuals without known diabetes had dysglycaemia, which was not supported after source reconciliation.

The high frequency of dysglycaemia is clinically important because it identifies a group requiring confirmatory testing, cardiovascular risk assessment and structured follow-up. However, these findings should not be interpreted as confirmed new diabetes diagnoses. South African primary-care guidance requires confirmatory testing in asymptomatic individuals with abnormal screening results, and single-event HbA1c screening cannot by itself establish a definitive diagnosis.^5^ The most appropriate interpretation is therefore that the audit identified a substantial burden of screen-positive glycaemic abnormality, rather than confirmed incident diabetes.

These findings are consistent with the broader African diabetes burden, where delayed detection and undiagnosed diabetes remain major public-health challenges.^1,3,4^ Opportunistic HbA1c screening in healthcare-engaged populations may therefore be useful as a case-finding entry point, but its clinical value depends on reliable confirmatory testing, counselling, treatment initiation or intensification and documented linkage to longitudinal care.

### Glycaemic control among participants with known diabetes

Overall glycaemic target attainment was incomplete. Across the 5-year series, 37/98 (37.8%; 95% CI 28.8–47.6) participants with known diabetes achieved HbA1c < 7.0%. Annual point estimates were below 50% in four of the five audit years, although CIs were wide because annual denominators were small.

The 2022 glycaemic-control estimate was an outlier. Although 15/18 participants with known diabetes were at target, the CI was wide, and the denominator was small. The annual report described newly available point-of-care HbA1c devices and multidisciplinary support, including trained nursing staff, dietetic input and podiatry involvement, but the audit design cannot determine whether the estimate reflects true clinical improvement, selection of more stable participants, sampling variation or data-capture effects. This result should therefore be interpreted cautiously and not as proof of sustained improvement in glycaemic control.

The overall pattern remains concerning. Persistent suboptimal glycaemic control increases the risk of microvascular complications, cardiovascular disease and premature morbidity.^12,13^ In a public-sector setting, poor control may reflect interacting patient-level and system-level factors, including therapeutic inertia, medication availability, dietary constraints, transport barriers, competing comorbidities and limited structured follow-up. The data support the need for screening activities to be linked to active treatment review rather than functioning as isolated awareness events.

### Hypertension and blood pressure control

Known hypertension was common, recorded in 149/334 (44.6%; 95% CI 39.4–50.0) participants in years with available hypertension-status data. Among participants with known hypertension and reconcilable BP-control data, only 41/107 (38.3%; 95% CI 29.7–47.8) achieved BP < 140/90 mmHg. This confirms a major gap in cardiometabolic risk-factor control.

Hypertension is a leading modifiable risk factor for stroke, heart failure, chronic kidney disease and premature cardiovascular mortality.^2,6^ Its coexistence with diabetes further amplifies vascular and renal risk.^6,13^ The low BP-control estimates in this screening series suggest that opportunistic BP measurement should be paired with protocolised repeat measurement, medication review, adherence assessment and follow-up planning.

Interpretation of BP findings must also consider measurement limitations. In the audit protocol, repeat BP measurement was performed only when the first reading was elevated, and the lower value was retained. This differs from preferred repeated-measurement or averaged-measurement practice and may introduce measurement error or regression-to-the-mean effects. Future screening should standardise BP measurement more rigorously.

### RVD-associated cardiometabolic multimorbidity

The 2025 audit incorporated recorded RVD status, allowing exploratory assessment of RVD-associated cardiometabolic overlap. RVD-associated multimorbidity was observed, including five complete-case participants with recorded RVD, known diabetes and known hypertension. However, subgroup estimates were imprecise and did not establish equivalence or a statistically significant difference between RVD-positive and RVD-negative participants.

This wording replaces the earlier unsupported claim that dysglycaemia and hypertension were ‘not less prevalent’ among RVD-positive participants. The revised interpretation is more cautious and better aligned with the available data. In the RVD-stratified complete-case analysis, known diabetes, known hypertension, dysglycaemia and BP control all had wide CIs. Therefore, the findings demonstrate observed overlap but do not support strong comparative claims.

Despite this limitation, the inclusion of RVD status remains clinically relevant. People living with HIV are increasingly affected by non-communicable diseases as survival improves with antiretroviral therapy.^7,8^ Chronic immune activation, inflammation, antiretroviral-associated metabolic effects, dyslipidaemia, insulin resistance and vascular ageing may contribute to cardiometabolic risk.^7,8,9,10,11^ These mechanisms support integrated HIV–NCD screening and follow-up models, particularly in high HIV-burden settings such as KwaZulu-Natal.

### Implications for practice and health systems

The findings suggest that annual awareness-day screening can identify abnormal glycaemic results, uncontrolled diabetes, uncontrolled hypertension and multimorbidity clusters. However, screening alone is insufficient. The clinical impact of these events depends on what happens after the abnormal result is identified.

A more robust model would include standardised data capture, repeated BP measurements, predefined referral pathways, confirmatory glucose testing, clinician review for treatment intensification, patient education and documented linkage to primary-care or specialist follow-up. Integration with HIV services is also important, because patients with RVD may already be engaged in chronic care and could benefit from embedded diabetes and hypertension screening within existing service platforms.

### Strengths and limitations

A strength of this study is that it provides serial cross-sectional data over five annual screening cycles within the same public-sector tertiary-hospital environment. The use of source-reconciled denominators, Wilson CIs, continuous variable summaries and RVD-stratified analysis improves the reliability and transparency of the revised analysis. The inclusion of RVD status in 2025 adds important local information on cardiometabolic overlap in a high HIV-burden setting.

Several limitations must be acknowledged. The sample was voluntary, non-random, healthcare-engaged and drawn from outpatient attendees and staff volunteers. The staff-patient mix was not consistently retained, repeat attendance across years could not be assessed, and the findings should not be interpreted as community prevalence estimates. Annual sample sizes were modest, some variables were missing, and age eligibility was not consistently documented across all source files. The 2024 BP-control estimate could not be reliably reconciled because the source report used conflicting BP thresholds and subgroup percentages. HbA1c-based screening results were not confirmed with repeat testing, fasting plasma glucose or oral glucose tolerance testing. RVD status was based on recorded or self-reported status, and no audit-day HIV testing was documented. The design was descriptive and cross-sectional, so causal inference and longitudinal outcome assessment were not possible.

## Conclusion

This 5-year series demonstrates a substantial burden of abnormal glycaemic screening results and incomplete cardiometabolic target attainment in a selected hospital-based screening sample. RVD-associated overlap with known diabetes and hypertension was observed, but subgroup comparisons were imprecise. Future screening should incorporate standardised repeated measurements, confirmatory testing pathways, complete electronic data capture and documented linkage to care.

## References

[1] International Diabetes Federation. IDF diabetes atlas. 10th ed. Brussels: International Diabetes Federation; 2021.

[2] World Health Organization. Global report on hypertension: The race against a silent killer. Geneva: World Health Organization; 2023.

[3] Mbanya JC, Motala AA, Sobngwi E, Assah FK, Enoru ST. Diabetes in sub-Saharan Africa. Lancet. 2010;375(9733):2254–2266. 10.1016/S0140-6736(10)60550-8 20609971

[4] Saeedi P, Petersohn I, Salpea P, et al. Global and regional diabetes prevalence estimates for 2019 and projections for 2030 and 2045: Results from the International Diabetes Federation Diabetes Atlas, 9th edition. Diabetes Res Clin Pract. 2019;157:107843. 10.1016/j.diabres.2019.107843 31518657

[5] National Department of Health, South Africa. Primary healthcare standard treatment guidelines and essential medicines list: Chapter 9, endocrine conditions. Pretoria: National Department of Health; 2025.

[6] Seedat YK, Rayner BL, Veriava Y. South African hypertension practice guideline 2014. Cardiovasc J Afr. 2014;25(6):288–294. 10.5830/CVJA-2014-062 PMC4327181 25629715

[7] Lake JE, Currier JS. Metabolic disease in HIV infection. Lancet Infect Dis. 2013;13(11):964–975. 10.1016/S1473-3099(13)70271-8 24156897

[8] Deeks SG, Phillips AN. HIV infection, antiretroviral treatment, ageing, and non-AIDS-related morbidity. BMJ. 2009;338:a3172. 10.1136/bmj.a3172 19171560

[9] Brown TT, Glesby MJ. Management of the metabolic effects of HIV and HIV drugs. Nat Rev Endocrinol. 2012;8(1):11–21. 10.1038/nrendo.2011.151 PMC3371609 21931374

[10] Nduka CU, Stranges S, Kimani PK, Sarki AM, Uthman OA. Is there sufficient evidence for a causal association between antiretroviral therapy and diabetes in HIV-infected patients? A meta-analysis. Diabetes Metab Res Rev. 2017;33(6):e2902. 10.1002/dmrr.2902 28437854

[11] Nduka CU, Stranges S, Sarki AM, Kimani PK, Uthman OA. Evidence of increased blood pressure and hypertension risk among people living with HIV on antiretroviral therapy: A systematic review with meta-analysis. J Hum Hypertens. 2016;30(6):355–362. 10.1038/jhh.2015.97 26446389

[12] American Diabetes Association Professional Practice Committee. 2. Diagnosis and classification of diabetes: Standards of Care in Diabetes – 2024. Diabetes Care. 2024;47(Suppl 1):S20–S42. 10.2337/dc24-S002 PMC10725812 38078589

[13] SEMDSA Type 2 Diabetes Guidelines Expert Committee. SEMDSA 2017 guidelines for the management of type 2 diabetes mellitus. J Endocrinol Metab Diabetes S Afr. 2017;22(1 Suppl 1):S1–S196.

